# Genomic characterization of *Escherichia coli* LCT-EC001, an extremely multidrug-resistant strain with an amazing number of resistance genes

**DOI:** 10.1186/s13099-019-0298-5

**Published:** 2019-05-21

**Authors:** Xuelin Zhang, Saisong Xiao, Xuege Jiang, Yun Li, Zhongyi Fan, Yi Yu, Peng Wang, Diangeng Li, Xian Zhao, Changting Liu

**Affiliations:** 1 Respiratory Diseases Department, The Second Medical Center of PLA General Hospital, Beijing, 100853 China; 2 Department of Anesthesiology, Dongzhimen Hospital Beijing University of Chinese Medicine, Beijing, 100700 China; 3 Respiratory Diseases Department, The Eighth Medical Center of PLA General Hospital, Beijing, 100091 China; 4 Hyperbaric Oxygen Department, The First Medical Center of PLA General Hospital, Beijing, 100853 China

**Keywords:** *Escherichia coli*, Antibiotic resistance, High-throughput sequencing, ESBL, KPC

## Abstract

**Background:**

Multidrug resistance is a growing global public health threat with far more serious consequences than generally anticipated. In this study, we investigated the antibiotic resistance and genomic traits of a clinical strain of *Escherichia coli* LCT-EC001.

**Results:**

LCT-EC001 was resistant to 16 kinds of widely used antibiotics, including fourth-generation cephalosporins and carbapenems. In total, up to 68 determinants associated with antibiotic resistance were identified, including 8 beta-lactamase genes (notably producing ESBLs and KPCs), 31 multidrug efflux system genes, 6 outer membrane transport system genes, 4 aminoglycoside-modifying enzyme genes, 10 two-component regulatory system genes, and 9 other enzyme or transcriptional regulator genes, covering nearly all known drug-resistance mechanisms in *E. coli*. More than half of the resistance genes were located close to mobile genetic elements, such as plasmids, transposons, genomics islands, and insertion sequences. Phylogenetic analysis revealed that this strain may have evolved from *E. coli* K-12 but is a completely new MLST type.

**Conclusions:**

Antibiotic resistance was extremely severe in *E. coli* LCT-EC001, mainly due to mobile genetic elements that allowed the gain of a large quantity of resistance genes. The antibiotic resistance genes of *E. coli* LCT-EC001 can probably be transferred to other bacteria. To the best of our knowledge, this is the first report of a strain of *E. coli* which has such a large amount of antibiotic resistance genes. Apart from providing an *E. coli* reference genome with an extremely high multidrug-resistant background for future analyses, this work also offers a strategy for investigating the complement and characteristics of genes contributing to drug resistance at the whole-genome level.

**Electronic supplementary material:**

The online version of this article (10.1186/s13099-019-0298-5) contains supplementary material, which is available to authorized users.

## Background

According to the World Health Organization (WHO) report ‘Antimicrobial resistance: global report on surveillance 2014’, multidrug resistance is a growing global public health threat with far more serious consequences than generally anticipated. Out of the WHO member states, 50% reported that *E. coli* isolated from within these states was resistant to third-generation cephalosporins and fluoroquinolones—the best antibiotics available for treating multidrug-resistant bacteria. In February 2017, the WHO published its first ever list of antibiotic-resistant “priority pathogens”—a catalogue of 12 families of bacteria that pose the greatest threat to human health. *E. coli* was defined as one of the most critical multidrug-resistant bacteria, which were considered to have built-in abilities to find new ways to resist treatment and pass along genetic material that allows other bacteria to become drug-resistant as well. It is widely accepted that infections caused by antibiotic-resistant bacteria burden healthcare resources and increase the risk of poor clinical outcomes for patients. Global estimates suggest that more than 700,000 people per year die from drug-resistant infections [1]. It is predicted that antibiotic-resistant infections will kill ~ 10 million people per year by 2050, costing the global economy ~ $100 trillion [2]. The seriousness of this situation was surmised in the WHO report: ‘A post antibiotic era, in which common infections and minor injuries can kill, is instead a very real possibility for the 21st century’.

Revealing the mechanisms underlying drug resistance in bacterial pathogens is crucial in infection disease control and management. With significant progress in high-throughput sequencing and bioinformatics analysis of pathogens, whole-genome sequencing has become more accessible for the identification and tracking of multidrug-resistance (MDR) microorganisms in hospitals and communities [3]. In this study, we isolated *E. coli* strain LCT-EC001 from a 78-year-old male patient with several health issues, including diabetes, hypertension and chronic obstructive pulmonary disease, who had received long-term therapy with multiple drugs. The drug resistance of *E. coli* strain LCT-EC001 was tested, and whole-genome sequencing was conducted to understand the genetic elements contributing to antibiotic resistance. This work contributes a clinically isolated drug-resistant *E. coli* strain as a valuable reference for future studies and presents a strategy for the comprehensive analysis of drug resistance at the whole-genome level.

## Methods

### Bacterial isolation and culture conditions

An *E. coli* isolate (designated LCT-EC001) was obtained from the sputum of a 78-year-old male patient who had several health issues (diabetes, hypertension and chronic obstructive pulmonary disease) and had received multidrug therapy over a long time period. The bacterium was inoculated in Brain Heart Infusion (Oxoid, UK) medium at 37 °C.

### Antibiotic susceptibility test

The antibiotic susceptibility profile was tested using a VITEK 2 Compact System (bioMerieux Inc., USA) according to the manufacturer’s instructions as previously reported [4]. 17 kinds of antibiotics tested are as follows: ampicillin, cefazolin, ampicillin/sulbactam, cefotetan, ceftriaxone, cefepime, ceftazidime, aztreonam, ertapenem, imipenem, amikacin, gentamicin, tobramycin, levofloxacin, ciprofloxacin, trimethoprim/sulfa, and nitrofurantoin.

### High-throughput sequencing and assembly

Isolation of genomic DNA was carried out using the cetyltrimethylammonium bromide (CTAB) method. Total DNA obtained was subjected to quality control by agarose gel electrophoresis and quantified by Qubit [5]. The genome of *E. coli* strain LCT-EC001 was sequenced with MPS (massively parallel sequencing) Illumina technology. Two DNA libraries were constructed: a paired-end library with an insert size of 500 bp and a paired-end library with an insert size of 5 kb. The 500 bp library and the 5 kb library were sequenced using an Illumina HiSeq 2000 platform (Illumina, USA). Quality control of the two paired-end library reads was performed using readfq (version 10) program [6] with the following steps: (1) Eliminate reads once its low quality nucleotide bases (Q-value ≤ 38) exceeding the threshold (40 bp by default), (2) Eliminate the reads containing Ns in the reads greater than the threshold (10 bases by default), (3) Eliminate reads whose overlap with the adapter exceeding the threshold (15 bp by default), and (4) Filter duplicates to keep only one copy of the totally same reads. For a library of 500 bp, 6.19% of reads were filtered, while 8.48% of reads were filtered for a library of 5 kb. The filtered reads were assembled by SOAPdenovo [7] to generate scaffolds. The parameters used for assembly were as follows: SOAPdenovo all -F -K 107 -k 107. All reads were used for further gap closure by using GapCloser (version 1.12) [8] with default parameters.

### Gene prediction, annotation and protein classification

Gene prediction was performed on the LCT-EC001 genome assembly by GeneMarkS [9] with an integrated model that combined the GeneMarkS generated (native) and heuristic model parameters. Gene annotation was performed with a BLASTp [10] search (E-value less than 1·e^−5^, minimal alignment length percentage larger than 40%) against 4 databases in a standalone environment. The databases are KEGG (Kyoto Encyclopedia of Genes and Genomes, v2016.4) [11], COG (Clusters of Orthologous Groups, v2015.12) [12], GO (Gene Ontology, v2014.10) [13], and ncRNA (noncoding RNA database, tRNA: v1.3.1, rRNA: v1.2, and sRNA: v2013.8) [14–16]. A genome overview was created with Circos [17] to show annotation information. In addition, genomic islands (GIs), prophages, repeat regions, transfer elements, plasmids, and insertion sequences elements (IS elements) in LCT-EC001 were analyzed. Repetitive sequences were predicted using RepeatMasker [18]. Tandem repeats were analyzed using Tandem Repeat Finder (TRF) [19]. PHAST [20] was used for prophage prediction. IslandPath-DIOMB [21] was used to predict genomic islands and horizontal gene transfer by examining features such as dinucleotide sequence composition bias and the presence of mobility genes.

### Phylogenetic analysis and multilocus sequence typing (MLST)

The genome datasets of the other 62 *E. coli* strains were compared with the genome of LCT-EC001 for SNP detection by using MUMmer with default settings (version 3.22). Then, the repeat regions of LCT-EC001 were detected by self-blast (choosing BLASTn parameter with blastall, using BLAST v2.2.23), TRF and RepeatMasker. After that, SNPs located in the repeat region were filtered. Based on the location array of SNPs, a phylogenetic tree was generated using the neighbor-joining method with 1000 bootstraps via MEGA6. MLST was performed with the web tool at http://cge.cbs.dtu.dk/services/MLST/, using the assembled genome. By comparing the sequences of seven housekeeping genes (ADK,FUMC,GYRB,ICD,MDH,PURA,RECA) in LCT-EC001 with that in the database, the MLST type was analyzed.

### Analysis of antibiotic resistance genes

A BLASTp [10] search (E-value less than 1·e^−5^, minimal alignment length percentage larger than 40%) was performed against 3 databases for drug resistance analysis. The databases are ARDB (Antibiotic Resistance Genes Database), CARD and ARG-ANNOT (Antibiotic Resistance Gene-ANNOTation). Then, the identified sequences were all BLAST searched online (https://blast.ncbi.nlm.nih.gov/Blast.cgi) to match genes in NCBI. The identified resistance genes were further verified by PCR and Sanger sequencing. Location relationships between these identified genes and genomic islands, prophages, repeat regions, transfer elements, plasmids, and IS elements were analyzed.

## Results and discussion

### Strain LCT-EC001 is resistant to most clinical antibiotics

We tested the susceptibility of *E. coli* strain LCT-EC001 to 17 kinds of widely used antibiotics with the VITEK 2 Compact System in triplicate. Our findings showed that *E. coli* strain LCT-EC001 was resistant to 16 kinds of antibiotics, including fourth-generation cephalosporins (cefepime) and carbapenems (ertapenem and imipenem), and was only sensitive to amikacin, indicating that it is a severely multidrug-resistant bacterium. However, extended spectrum β-lactamases (ESBL) were negatively detected. The results are shown in Table 1.

**Table 1:** Antimicrobial susceptibility profile of *E. coli* strain LCT-EC001

Classification	Antibiotic	MIC (μg/ml)	Sensitivity
Beta-lactam antibiotics	Ampicillin	≥ 32	R
	Cefazolin	≥ 64	R
	Ampicillin/sulbactam	≥ 32	R
	Cefotetan	≥ 64	R
	Ceftriaxone	≥ 64	R
	Ceftazidime	≥ 64	R
	Cefepime	≥ 64	R
	Aztreonam	≥ 64	R
	Ertapenem	≥ 8	R
	Imipenem	4	R
	ESBL	Neg	
Aminoglycoside antibiotics	Amikacin	≤ 2	S
	Gentamicin	≥ 16	R
	Tobramycin	≥ 16	R
Quinolone antibiotics	Levofloxacin	≥ 8	R
	Ciprofloxacin	≥ 4	R
Sulfonamides	Trimethoprim/sulfa	≥ 320	R
Nitrofurans	Nitrofurantoin	≥ 64	I

*R* resistant, *S* sensitive, *I* intermediate

Normally, *E. coli* colonizes the intestines of humans and other animals [22]. However, it is a frequent cause of community and hospital-acquired infections, such as those of the urinary tract, bloodstream, abdomen, skin and soft tissues under certain circumstances [23]. This bacterium also causes pneumonia, neonatal meningitis and food-borne infections on a global scale [24]. It is well accepted that antimicrobial resistance is related to widespread antibiotic use, especially their inappropriate use in humans and other animals, as well as in the food industry [25]. With the increasing incidence of multidrug-resistant organisms, antibiotic resistance has now become a serious global public health problem.

### Genomic features of the strain LCT-EC001

**Fig. 1:**
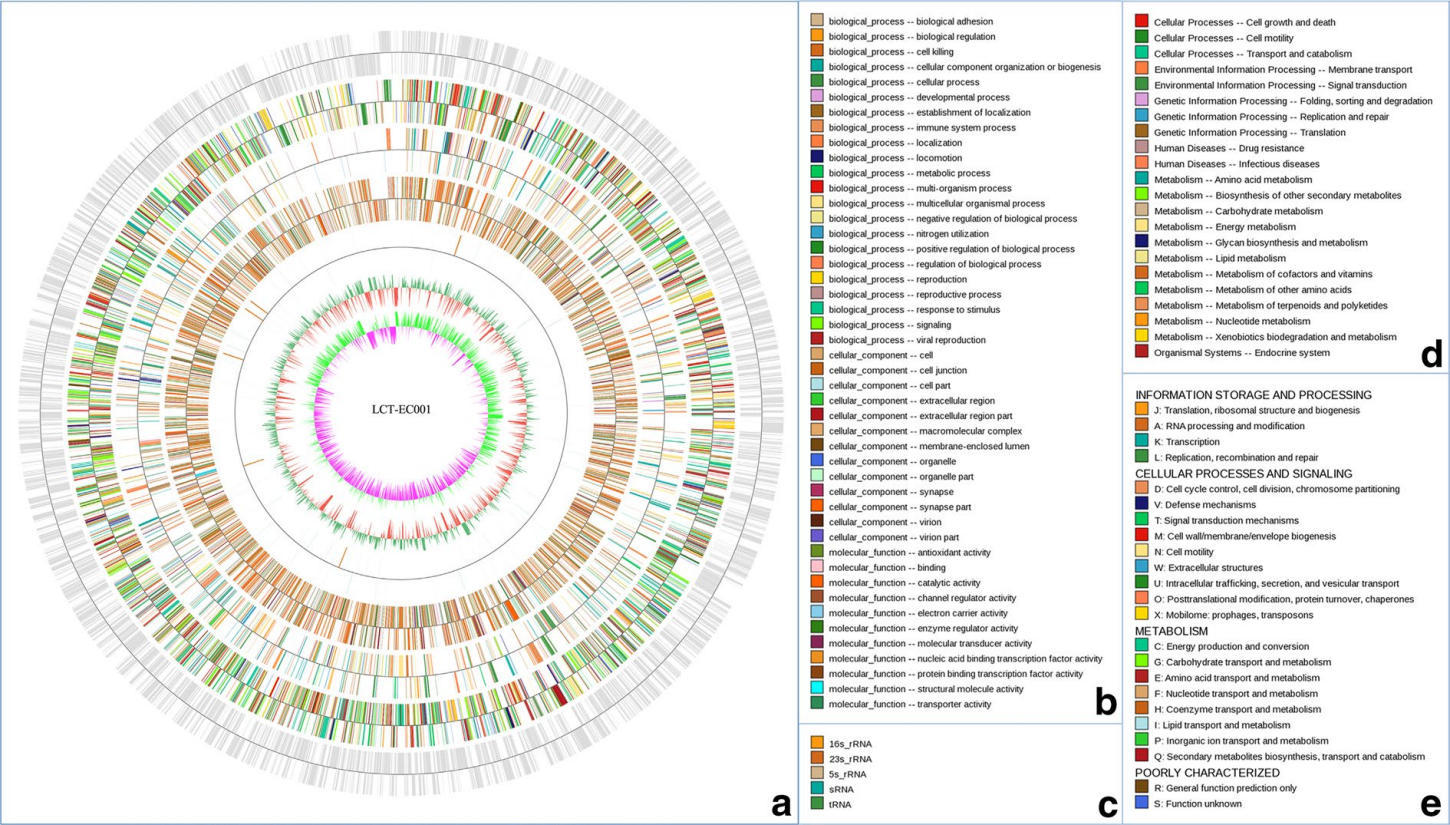
Genomic map of *E. coli* strain LCT-EC001. From outer to inner in **a**, the first circle shows the identified genes in LCT-EC001. The 2nd–4th circles show the COG, KEGG, and GO functions of these identified genes, respectively, and each color represents a function classification in which details are annotated in **e**, **d**, and **b**, respectively. The 5th circle shows the ncRNA results, and each color represents a classification in which details are annotated in **c**. For these five circles, the outer side of each represents a positive strand, while the inner side represents a negative strand. The 6th circle shows the GC contents. The 7th circle represents the LCT-EC001 GC-skew distribution. GC-skew = (G − C)/(G + C); purple and green indicate positive and negative values, respectively

An illustration of the genomic contents in the genome of *E. coli* strain LCT-EC001 is shown in Fig. 1. The final assembled genome consisted of 17 scaffolds with a total length of 5,198,242 bp and a mean GC content of 50.79%. The gene annotation included 5013 protein coding sequences (CDSs) accounting for 86.61% of the genome (Table 2), 84 tRNA (transfer RNA) fragments, 65 snRNA (small nuclear RNA) genes, 7 copies of 5S rRNA (ribosomal RNA), 6 copies of 16S rRNA, 6 copies of 23S rRNA (Additional file 1: Table S1), 17,031 bp of interspersed repeat sequences and 31,219 bp of tandem repeat sequences (Additional file 2: Table S2). A total of 69.18% of the gene distribution in the GO database is shown in Additional file 3: Table S3, 78.04% in the COG database shown in Additional file 4: Table S4, and 65.93% in the KEGG database shown in Additional file 5: Table S5.

**Table 2:** The genome summary of *E. coli* strain LCT-EC001

Genome size (bp)	5,198,242
Scaffold number	17
N50 length (bp)	4,259,009
GC content (%)	50.79
Gene number	5013
Gene average length (bp)	898
GC content of genes (%)	50.79

### Phylogenetic tree and MLST analysis of LCT-EC001

**Fig. 2:**
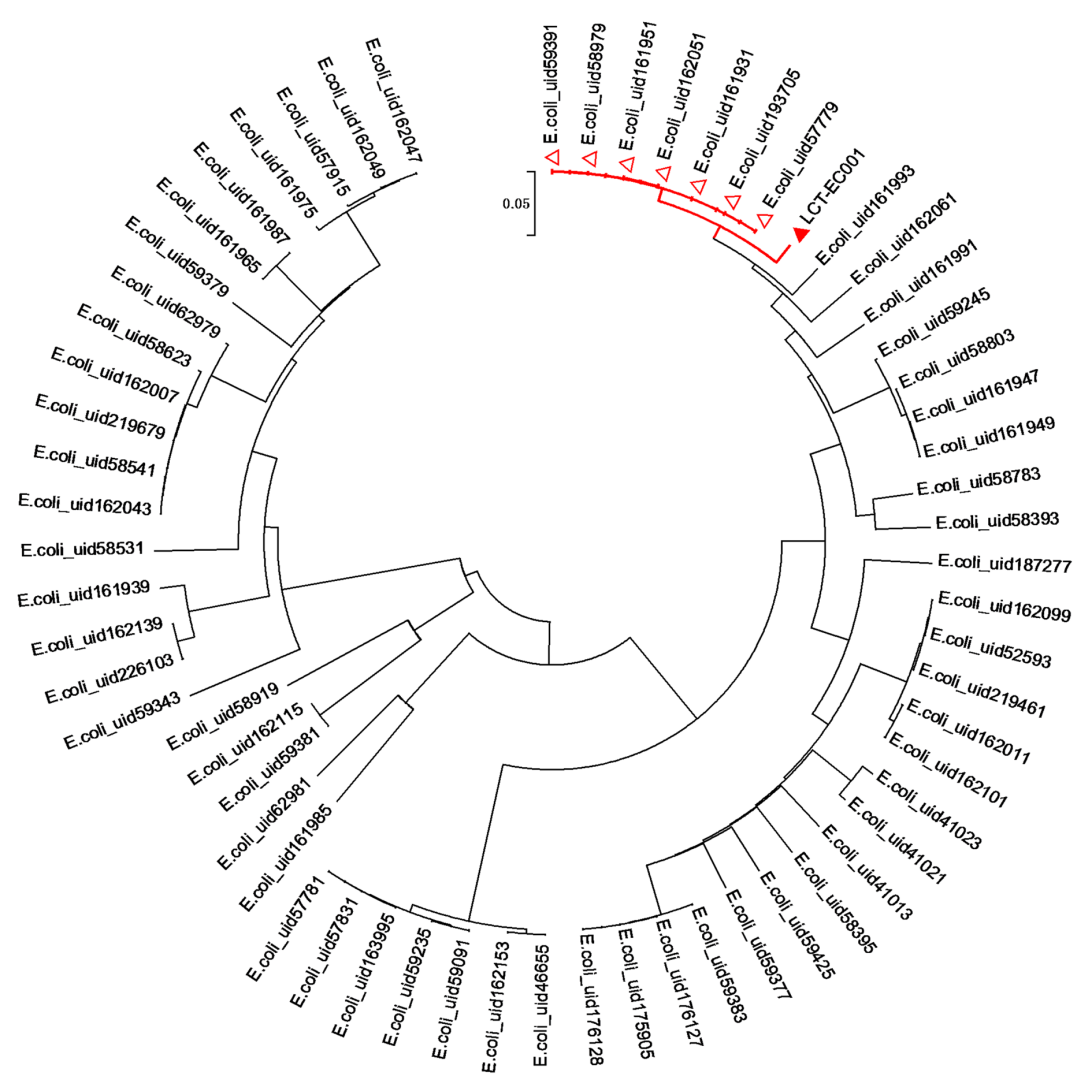
Evolutionary relationships between LCT-EC001 and other *E. coli* strains. Sixty-two strains of *E. coli* with complete genomes from NCBI were used for phylogenetic analysis. The phylogenetic tree was deduced by neighbor-joining. From the results, we found that the strain LCT-EC001 was close to the lineage of *E. coli* K-12 (represented with “Δ”). The names of the *E. coli* strains were composed of *E. coli* and the NCBI uid

To interpret the evolution of such an extreme multidrug-resistant *Escherichia coli* isolate, a selection of 62 *E. coli* complete genomes (1 chromosome) downloaded from NCBI was used to map phylogenetic trees by using neighbor-joining. All samples except LCT-EC001 were named as *E. coli* plus the NCBI uid. The results showed that LCT-EC001 was most closely related to *E. coli* K-12, which is mostly used in laboratories (Fig. 2), indicating that LCT-EC001 may have evolved. MLST analysis showed that the seven housekeeping genes in LCT-EC001 were ADK10, FUMC11, GYRB4, ICD8, MDH8, PURA13, and RECA2. However, no available MLST type could match that of LCT-EC001, revealing that this strain was a completely new type.

### Analysis of the complement of antibiotic resistance genes

**Fig. 3:**
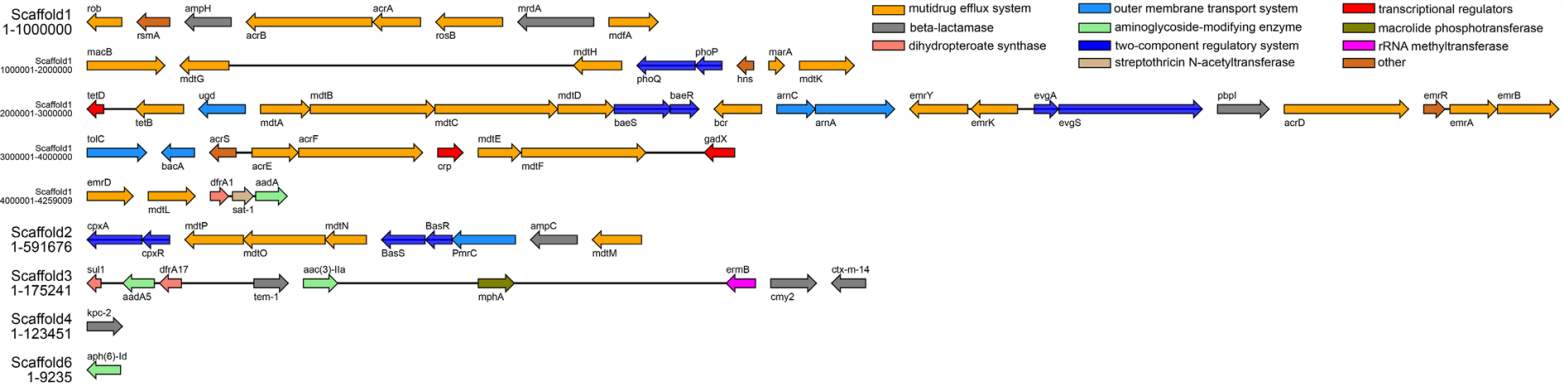
Antibiotic resistance genes in LCT-EC001. Resistance genes matched in the ARDB, CARD, or ARG-ANNOT database were identified in scaffolds 1–6, mainly in scaffold1. As scaffold1 is too long, it is shown in five sections with 1 Mbp separation. Genes of the same function classification are shown in the same color

To understand the basis of antibiotic resistance in *E. coli* strain LCT-EC001, we carried out sequence alignments with the ARDB database, CARD database and ARG-ANNOT database. A total of 68 determinants associated with antibiotic resistance were identified, with a length range of 348–3594 bp, and mean length of 1305 bp (Additional file 6: Table S6). All those determinants were matched to genes in NCBI with similarity of at least 99%, then further named and classified according to the matched gene information, including 8 beta-lactamase genes, 31 multidrug efflux system genes, 6 outer membrane transport system genes, 4 aminoglycoside-modifying enzyme genes, 10 two-component regulatory system genes, and 9 other enzyme or transcriptional regulator genes (Fig. 3). PCR and Sanger sequencing were further used to confirm that all the genes did exist in *E. coli* strain LCT-EC001. Beta-lactamases are enzymes produced by bacteria that provide resistance to β-lactam antibiotics such as penicillins, cephalosporins, and cephamycins by breaking the antibiotics’ structure, a four-atom ring known as a β-lactam. Among the 8 beta-lactamase genes, 2 were the extended-spectrum β-lactamase (ESBL) genes Tem-1 and CTXM-14, and 1 was the *Klebsiella pneumoniae* carbapenemase (KPC) gene KPC-2. ESBLs can hydrolyze extended-spectrum cephalosporins, including cefotaxime, ceftriaxone, and ceftazidime, as well as the oxyimino-monobactam aztreonam. Thus, ESBLs confer multiresistance to these antibiotics and related oxyimino-beta lactams, which play an important role in antibiotic resistance in *E. coli*. KPC is another key enzyme in MDR, due to its ability to hydrolyze a broad variety of β-lactams, including carbapenems, cephalosporins and penicillins [26]. Interestingly, ESBL gene were not detected by VITEK 2 Compact System, highlighting its flaws in clinical setting.

The drug resistance genes in LCT-EC001 covered nearly all known drug-resistance mechanisms in *E. coli*. Of these genes, 34 genes were detected from the ARDB database, 61 genes were detected from the CARD database, and 19 genes were detected from the ARG-ANNOT database (Additional file 7: Table S7). In addition, 6 of these genes were located in genome islands, 11 genes were located in plasmids, 3 genes were near transposons, 14 genes were near insertion sequences, and no genes were related to prophages or repeat regions (Additional file 7: Table S7). A more concerning problem is that antibiotic resistance traits in bacteria can transfer between each other, regardless of their genus [27], via mobile genetic elements (MGEs) such as plasmids [28], insertion sequences [29], integrons/transposons [30], and chromosomal fragments (including resistance islands) [31]. A plasmid is a kind of extrachromosomal DNA molecule with the ability to autonomously replicate. A plasmid can harbor genes encoding β-lactams, even carbapenemases or extended-spectrum β-lactamases, and aminoglycosides [32] and genes producing antibiotic-target protecting proteins, antibiotic-modifying enzymes or multidrug efflux pumps [33]. Plasmids can also acquire mobile genetic elements by encoding endonucleases/methylase restriction systems [34]. Furthermore, plasmids can move from one bacterial cell to another by conjugal transfer [34], playing a vital role in the spread of resistance determinants among bacteria. An insertion sequence (IS) is an important MGE that widely exists in bacterial genomes, usually with a length of 0.6–2.0 kb [35]. IS elements can help resistance genes to transfer between and within bacteria [36] and can upregulate downstream resistance genes [37]. Integrons are another MGE responsible for the emergence and spread of antibiotic resistance genes, including β-lactamases, aminoglycosides, and fluoroquinolones [38]. Transposons, like plasmids, have the potential to transfer horizontally or vertically among pathogens, driving the development of antibiotic resistance [39]. A genomic island (GI), usually with a size of 4.5–600 kb and generated by lateral gene transfer (LGT), is a large continuous genomic region. In addition, GIs can carry tens to hundreds of genes, often important for bacterial evolution, such as antibiotic resistance [40].

It is worth mentioning that our genome is a draft genome comprising 18 contigs, which means there are 17 gaps of sequence missed and other drug-resistant genes that may not have been identified.

## Additional files

**Additional file 1.** Gene annotation of LCT-EC001.

**Additional file 2.** Repeat sequences of LCT-EC001.

**Additional file 3.** Gene distribution of LCT-EC001 in the GO database.

**Additional file 4.** Gene distribution of LCT-EC001 in the COG database.

**Additional file 5.** Gene distribution of LCT-EC001 in the KEGG database.

**Additional file 6.** Gene length of 68 antibiotic resistance determinants in LCT-EC001.

**Additional file 7.** Location and database source of 68 antibiotic resistance determinants in LCT-EC001.

## References

[1] Ashiru-Oredope D, Hopkins S (2015). Antimicrobial resistance: moving from professional engagement to public action. J Antimicrob Chemother.

[2] Silva ON, de la Fuente-Núñez C, Haney EF, Fensterseifer IC, Ribeiro SM, Porto WF (2016). An anti-infective synthetic peptide with dual antimicrobial and immunomodulatory activities. Sci Rep..

[3] Quainoo S, Coolen JPM, van Hijum SAFT, Huynen MA, Melchers WJG, van Schaik W (2017). Whole-genome sequencing of bacterial pathogens: the future of nosocomial outbreak analysis. Clin Microbiol Rev.

[4] Li J, Liu F, Wang Q, Ge P, Woo PC, Yan J (2014). Genomic and transcriptomic analysis of NDM-1 *Klebsiella pneumoniae* in spaceflight reveal mechanisms underlying environmental adaptability. Sci Rep..

[5] Casaril Aline Etelvina, de Oliveira Liliane Prado, Alonso Diego Peres, de Oliveira Everton Falcão, Gomes Barrios Suellem Petilim, de Oliveira Moura Infran Jucelei, Fernandes Wagner de Souza, Oshiro Elisa Teruya, Ferreira Alda Maria Teixeira, Ribolla Paulo Eduardo Martins, de Oliveira Alessandra Gutierrez (2017). Standardization of DNA extraction from sand flies: Application to genotyping by next generation sequencing. Experimental Parasitology.

[6] Li H. Fast multi-line fasta/q reader in several programming languages. 2013. https://github.com/lh3/readfq.

[7] Li R, Zhu H, Ruan J, Qian W, Fang X, Shi Z (2010). De novo assembly of human genomes with massively parallel short read sequencing. Genome Res.

[8] Liu T, Zhu L, Zhang Z, Jiang L, Huang H (2018). Draft genome sequence of *Bacillus* sp. (2017) M13, a multidrug-resistant subclass B1 blaNDM-producing, spore-forming bacterium isolated from China. J Glob Antimicrob Resist..

[9] Besemer J, Lomsadze A, Borodovsky M (2001). GeneMarkS: a self-training method for prediction of gene starts in microbial genomes. Implications for finding sequence motifs in regulatory regions. Nucleic Acids Res.

[10] Rost B (1999). Twilight zone of protein sequence alignments. Protein Eng.

[11] Kanehisa M, Goto S, Hattori M, Aoki-Kinoshita KF, Itoh M, Kawashima S (2006). From genomics to chemical genomics: new developments in KEGG. Nucleic Acids Res..

[12] Tatusov RL, Fedorova ND, Jackson JD, Jacobs AR, Kiryutin B, Koonin EV (2003). The COG database: an updated version includes eukaryotes. BMC Bioinformatics.

[13] Ashburner M, Ball CA, Blake JA, Botstein D, Butler H, Cherry JM (2000). Gene ontology: tool for the unification of biology. The Gene Ontology Consortium. Nat Genet..

[14] Lowe TM, Eddy SR (1997). tRNAscan-SE: a program for improved detection of transfer RNA genes in genomic sequence. Nucleic Acids Res.

[15] Lagesen K, Hallin P, Rødland EA, Staerfeldt HH, Rognes T, Ussery DW (2007). RNAmmer: consistent and rapid annotation of ribosomal RNA genes. Nucleic Acids Res.

[16] Gardner PP, Daub J, Tate JG, Nawrocki EP, Kolbe DL, Lindgreen S (2009). Rfam: updates to the RNA families database. Nucleic Acids Res..

[17] Krzywinski M, Schein J, Birol I, Connors J, Gascoyne R, Horsman D (2009). Circos: an information aesthetic for comparative genomics. Genome Res.

[18] Saha S, Bridges S, Magbanua ZV, Peterson DG (2008). Empirical comparison of ab initio repeat finding programs. Nucleic Acids Res.

[19] Benson G (1999). Tandem repeats finder: a program to analyze DNA sequences. Nucleic Acids Res.

[20] Zhou Y, Liang Y, Lynch KH, Dennis JJ, Wishart DS (2011). PHAST: a fast phage search tool. Nucleic Acids Res..

[21] Bertelli C, Brinkman FSL (2018). Improved genomic island predictions with IslandPath-DIMOB. Bioinformatics.

[22] Qin J, Li R, Raes J, Arumugam M, Burgdorf KS, Manichanh C (2010). A human gut microbial gene catalogue established by metagenomic sequencing. Nature.

[23] Pennington THE (2014). *coli* O157 outbreaks in the United Kingdom: past, present, and future. Infect Drug Resist..

[24] Rohde H, Qin J, Cui Y, Li D, Loman NJ, Hentschke M (2011). Open-source genomic analysis of Shiga-toxin-producing *E. coli* O104:H4. N Engl J Med..

[25] Hawkey PM, Jones AM (2009). The changing epidemiology of resistance. J Antimicrob Chemother.

[26] Galdadas I, Lovera S, Pérez-Hernández G, Barnes MD, Healy J, Afsharikho H (2018). Defining the architecture of KPC-2 Carbapenemase: identifying allosteric networks to fight antibiotics resistance. Sci Rep..

[27] Tacconelli E, Sifakis F, Harbarth S, Schrijver R, van Mourik M, Voss A (2017). Surveillance for control of antimicrobial resistance. Lancet Infect Dis..

[28] Masud MR, Afroz H, Fakruddin M (2014). Prevalence of extended-spectrum β-lactamase positive bacteria in radiologically positive urinary tract infection. Springerplus..

[29] Zhong LL, Phan HTT, Shen C, Doris-Vihta K, Sheppard AE, Huang X (2018). High rates of human fecal carriage of mcr-1-positive multi-drug resistant *Enterobacteriaceae* isolates emerge in China in association with successful plasmid families. Clin Infect Dis.

[30] Subedi D, Vijay AK, Willcox M (2018). Overview of mechanisms of antibiotic resistance in *Pseudomonas aeruginosa*: an ocular perspective. Clin Exp Optom..

[31] Martínez JL, Baquero F (2014). Emergence and spread of antibiotic resistance: setting a parameter space. Ups J Med Sci..

[32] Carattoli A (2013). Plasmids and the spread of resistance. Int J Med Microbiol.

[33] Correia S, Poeta P, Hébraud M, Capelo JL, Igrejas G (2017). Mechanisms of quinolone action and resistance: where do we stand?. J Med Microbiol.

[34] Challacombe JF, Pillai S, Kuske CR (2017). Shared features of cryptic plasmids from environmental and pathogenic *Francisella* species. PLoS ONE.

[35] Schmitz-Esser S, Penz T, Spang A, Horn M (2011). A bacterial genome in transition–an exceptional enrichment of IS elements but lack of evidence for recent transposition in the symbiont *Amoebophilus asiaticus*. BMC Evol Biol.

[36] Bennett PM (2008). Plasmid encoded antibiotic resistance: acquisition and transfer of antibiotic resistance genes in bacteria. Br J Pharmacol.

[37] Figueiredo S, Poirel L, Papa A, Koulourida V, Nordmann P (2009). Overexpression of the naturally occurring blaOXA-51 gene in *Acinetobacter baumannii* mediated by novel insertion sequence ISAba9. Antimicrob Agents Chemother.

[38] Chen DQ, Jiang YT, Feng DH, Wen SX, Su DH, Yang L (2018). Integron mediated bacterial resistance and virulence on clinical pathogens. Microb Pathog..

[39] Rangasamy K, Athiappan M, Devarajan N, Samykannu G, Parray JA, Aruljothi KN (2017). Pesticide degrading natural multidrug resistance bacterial flora. Microb Pathog.

[40] Lu B, Leong HW (2016). Computational methods for predicting genomic islands in microbial genomes. Comput Struct Biotechnol J..

